# Identification of cell types from single cell data using stable clustering

**DOI:** 10.1038/s41598-020-66848-3

**Published:** 2020-07-23

**Authors:** Azam Peyvandipour, Adib Shafi, Nafiseh Saberian, Sorin Draghici

**Affiliations:** 1 Department of Computer Science, Wayne State University, Detroit, MI USA; 2 Department of Obstetrics and Gynecology, Wayne State University, Detroit, MI USA

**Keywords:** Gene expression analysis, Machine learning

## Abstract

Single-cell RNA-seq (scRNASeq) has become a powerful technique for measuring the transcriptome of individual cells. Unlike the bulk measurements that average the gene expressions over the individual cells, gene measurements at individual cells can be used to study several different tissues and organs at different stages. Identifying the cell types present in the sample from the single cell transcriptome data is a common goal in many single-cell experiments. Several methods have been developed to do this. However, correctly identifying the true cell types remains a challenge. We present a framework that addresses this problem. Our hypothesis is that the meaningful characteristics of the data will remain despite small perturbations of data. We validate the performance of the proposed method on eight publicly available scRNA-seq datasets with known cell types as well as five simulation datasets with different degrees of the cluster separability. We compare the proposed method with five other existing methods: RaceID, SNN-Cliq, SINCERA, SEURAT, and SC3. The results show that the proposed method performs better than the existing methods.

## Introduction

Recent advances in single-cell RNA-Seq (scRNASeq) techniques have provided transcriptomes of the large numbers of individual cells (single-cell gene expression data)^1–9^. In particular, analyzing the diversity and evolution of single cancer cells can enable the advances in early cancer diagnosis, and ultimately choosing the best strategy for cancer treatment^10–12^. Furthermore, one important analysis on scRNASeq is the identification of cell types that can be achieved by performing an unsupervised clustering method on transcriptome data^13–19^.

Clustering algorithms such as k-means and density-based spatial clustering of applications with noise (DBSCAN)^20^ can identify groups of cells given the single-cell gene expression data. However, clusters obtained by these algorithms might not be robust. Such algorithms require non-intuitive parameters^13^. For instance, given the number of clusters, k-means iteratively assigns data points (cells) to the nearest centroids (cluster center), and recomputes the centroids based on the predefined number of clusters. This algorithm starts with the randomly chosen centroids. Thus, the result of the algorithm depends on the number of clusters (in DBSCAN, the maximum distance between the two data points in the same neighborhood should be determined) and the number of runs.

Another challenge comes from the high dimensionality of data, known as “curse of dimensionality”. Identifying the accurate clusters of data points based on the measured distances between the pairs of data points may fail since those data points become more similar when they are represented in a higher dimensional space^13,21^. One approach to deal with the curse of high dimensionality is projecting data into a lower dimensional space, known as dimensionally reduction. In this approach, the data is represented in a lower dimensional space while the characteristic(s) (e.g similarities between the data points) of the original data is preserved. Several methods have used different techniques based on this concept (e.g. principal component analysis) to determine the cell types^22–26^. Another approach to deal with this challenge is feature selection, i.e. eliminating some of the features (genes) that are not informative^27^. In the following, we provide a brief overview of the related methods that identify the cell types based on the combination of approaches described above.

Methods SC3^28^ and Seurat^25^ use a combination of feature selection, dimensionality reduction, and clustering algorithms to identify the cell types. Authors of SC3 use a consensus clustering framework that combines clustering solutions obtained by the spectral transformations and k-means clustering based on the complete-linkage hierarchical clustering. They first apply a gene filtering approach on the single-cell gene expression data to remove rare and ubiquitous genes/transcripts. Next, they compute the distance matrices (distance between the cells) using the Euclidean, Pearson, and Spearman metrics. They transform the distance matrices using either principal component analysis (PCA)^29^, or by computing the eigenvectors of the associated graph Laplacian. Next, they perform a k-means clustering on the first *d* eigenvectors of the transformed distance matrices. Using the different k-means clustering results, they construct a consensus matrix that represents how often each pair of cells is clustered together. This consensus matrix is used as an input to a hierarchical clustering using a complete linkage and agglomeration strategies^30^. The clusters are inferred at the *k*-th level of hierarchy, where *k* is computed based on the Random Matrix Theory^31,32^. The accuracy of SC3 is sensitive to the number of eigenvectors (*d*), chosen for the spectral transformation. The authors report that SC3 performs well when *d* is between 4% and 7% of the number of cells. The main advantage of SC3 is its high accuracy in identification of cell types. However, it is not scalable^33^.

Seurat^25^ is a graph-based clustering method that projects the single cell expression data into the two-dimensional space using the t-distributed stochastic neighbor embedding (t-SNE) technique^34^. Then, it performs the DBSCAN method^20^ on the dimensionality-reduced single cell data. Seurat may fail to find the cell types in small datasets (low cell numbers)^28^. It is reported that this may be due to possible difficulties in estimating the densities when the number of data points is low.

RaceID^35^ determines the cell types by performing a k-means clustering algorithm. In this method, the gap statistics is used to choose the number of clusters. RaceID does not perform well when the data does not contain rare cell populations but it appears to be the preferred methods when the aim is identification of rare types^13,33,36,37^.

SNN-Cliq^17^ uses the shared nearest neighbor (SNN) concept, which considers the effect of the surrounding neighbor data points, to handle the high-dimensional data. The authors of SNN-Cliq compute the similarity between the pairs of data points (the similarity matrix) based on the Euclidean distance, referred as the primary similarity measure. Using the similarity matrix, they list the k-nearest neighbors (KNN) to each data point. They propose a secondary similarity measure that computes the similarity between two data points based on their shared neighborhoods. Consequently, an SNN graph is constructed based on the connectivity between the data points. Then, a graph-based clustering method is applied on the SNN graph in which nodes and weighted edges represent the data points and similarities between the data points, respectively. The main disadvantage of the graph-based methods such as SNN-Cliq is that scRNASeq data is not inherently graph-structured^13^. Therefore, the accuracy of these methods depends on the graph representation of scRNASeq data.

SINCERA^38^ performs a hierarchical clustering on the similarity matrix that is computed using the centered Pearson’s correlation. The average linkage approach is used as the default choice for the linkage. Consensus clustering^39,40^, tight clustering^41^ and ward linkage^42^ are provided as alternative clustering approaches. Users can choose a distance threshold or the number of clusters during the visual inspection when the hierarchical clustering is used for the cell cluster identification. SINCERA tends to identify many clusters which likely represent the same cell type^13^.

One way to identify robust clusters of cells is to resample the cells/genes and compare the original clusters with the ones that are obtained by resampling^43^. In the current paper, in order to explore the strength of a pattern (cluster of cells) in the data, we analyze the sensitivity of that pattern against small changes in the data. The data is resampled by replacing a certain number of data points with the noise points from a noise distribution. Our hypothesis is that if there is a strong pattern in data, it will remain despite small perturbations^44^. Here, we develop a stable subtyping (clustering) method that employs the t-distributed stochastic neighbor embedding (t-SNE)^34^ and k-means clustering to identify the cell types. We add noise and apply a bootstrap method^45,46^ to identify the stable clusters of cells. We use the Adjusted Rand Index (ARI)^47^, adjusted mutual information (AMI)^48,49^, and V-measure^50^ to evaluate the performance of the clustering result for datasets in which the true cell types are known. We compare the results of our method with five other methods: RaceID^35^, SNN-Cliq^17^, SINCERA^38^, SEURAT^25^, and SC3^28^ using 8 real datasets with known cell types and 5 simulated datasets. The results of the different methods show that the proposed method performs better than the five methods across different datasets.

## Materials and methods

**Figure 1:**
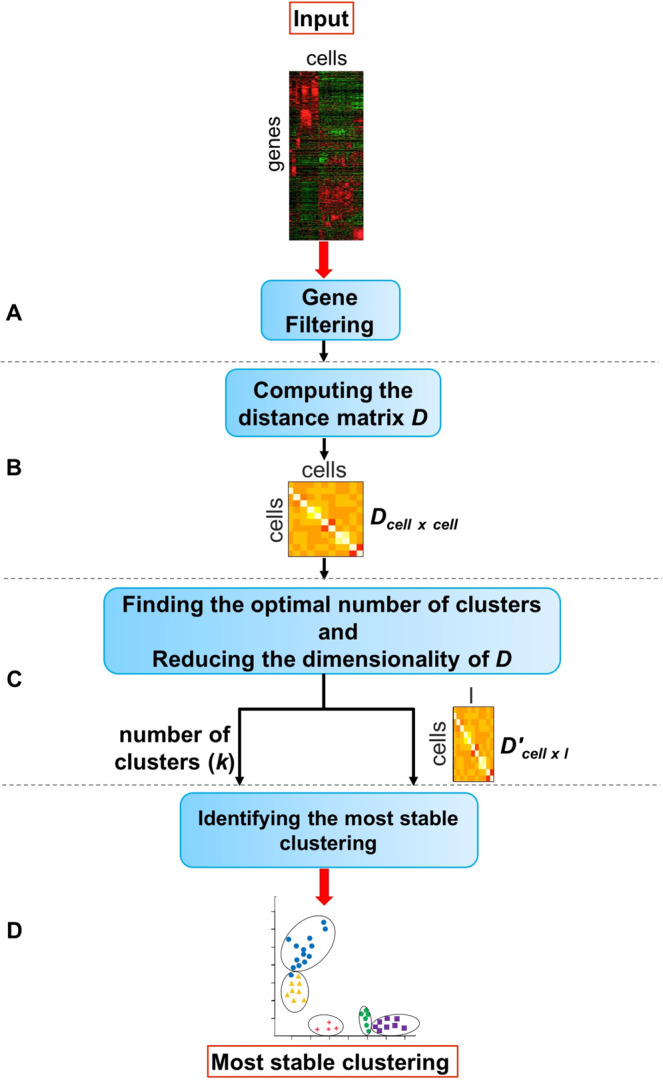
The overall workflow of the proposed method. Given the single cell gene expression matrix, **module (A)** eliminates the genes that are not expressed in any cell. Using the resulting matrix, **module (B)** computes the Euclidean distance between the cells. The output of this module is a distance matrix in which the rows and columns are the cells (*D*_*cell*×*cell*_). **Module (C)** reduces the dimensionality of the distance matrix using the t-distributed stochastic neighbor embedding (t-SNE) technique. In this module, an average silhouette method is employed to choose the optimal number of clusters *k*. Finally in **module (D)**, the lower-dimension distance matrix and the optimal number of clusters *k* obtained from **module (C)** are used as the input data to identify the most stable clustering of cells. Figure 2 shows the details of **module D**.

The goal of the proposed method is to identify the cell types present in a mixture of single cells. The input of the method is the single cell gene expression matrix (M_*gene*×*cell*_) in which rows represent the genes and columns represent the cells. In the following we provide more detail about the input data and different steps of the proposed framework. The overall approach is shown in Fig. 1.

### Data source

The eight publicly available scRNA-seq datasets as well as the five simulation datasets we used in our analysis are included in the Supplementary Materials. Among the eight real datasets, all but three (Klein^51^, Patel^52^, Treutlein^53^) are considered as’gold standard’ since the labels of the cells are known in a definitive way. Patel^52^ and Treutlein^53^ are referred as'silver standard’ by Kiselev *et al*.^28^ since their cell labels are determined based on the computational methods and the authors’ knowledge of the underlying biology.

We obtained the processed data from Hemberg lab's website (https://hemberg-lab.github.io/scRNA.seq.datasets). Hemberg *et al*.^54^ use the SingleCellExperiment Bioconductor S4 class^55^ to store the data, and the scater package^56^ for the quality control and plotting purposes. The normalized data is deposited as a SingleCellExperiment object (.RData file) and the cell type information is accessed in the cell_type1 column of the “colData” slot of this object. The gene expression values of the cells are organized as a matrix in which rows are cells and columns are the genes. In our analysis, genes (features) that are not expressed in any cells are removed. We did not filter any cell in this analysis.

### Gene filtering

As shown in Fig. 1A, we remove the genes/transcripts that are not expressed in any cell (expression value is zero in all cells). Such genes cannot provide useful information that can differentiate between cell types^57^. The result of performing the filtering method on the single cell gene expression matrix (M_*gene*×*cell*_) is used as the input to the second module of the proposed framework.

### Measuring the dissimilarity between the cells

The distance between the cells is calculated using the Euclidean metric (Fig. 1B). The output of this step is the distance (dissimilarity) matrix *D*_*cell*×*cell*_. We reduce the dimension of *D* by performing the t-distributed stochastic neighbor embedding (t-SNE)^34,58^, the nonlinear dimensionality reduction/visualization technique (Fig. 1C). We will refer to the output as *D*′_*cell*×*l*_, where 2 ≤ *l* ≤ *cell*. In this study, the number of dimensions is 2.

### Clustering

#### Identification of the optimal number of clusters

This section describes the third module of the proposed method (Fig. 1C). In this analysis, the t-SNE is repeatedly (n = 50) applied on the distance matrix *D*_*cell*×*cell*_ to obtain the dimensionality-reduced distance matrix *D*′_*cell*×*l*_. Each time, the optimal number of clusters is calculated based on the average silhouette method using the dimensionality reduced distance matrix *D*′. In order to find the optimal number of clusters *k*, the k-means clustering is applied on the *D*′ matrix using a range value (default = 2:20), and the *k* that maximizes the average silhouette measure is selected. Finally, the average of the selected numbers *k* across different repeats (*n* = 50) (rounded to the nearest integer) is considered as the final optimal number of clusters.

The silhouette evaluates the quality of that clustering based on how well its data points are clustered. A silhouette measure is assigned to each data point representing how close a data point is to its own cluster in comparison to other clusters. For each data point *i*, this measure is calculated as follows:

$${\rm{s}}({\rm{i}})=\frac{b(i)-a(i)}{max\{a(i),b(i)\}}$$

where *a*(*i*) is the average distance between the data point *i* and all other data points within the same cluster. *b*(*i*) is the smallest average distance of *i* to all points in any other cluster of which *i* is not a member. *s*(*i*) takes values from −1 to 1, where a high positive score shows that the given data point is well clustered (close to other points in its own cluster and far from points in the other clusters). Conversely, a high negative score shows that data point is poorly clustered.

#### k-means clustering based on the resampling method

**Figure 2:**
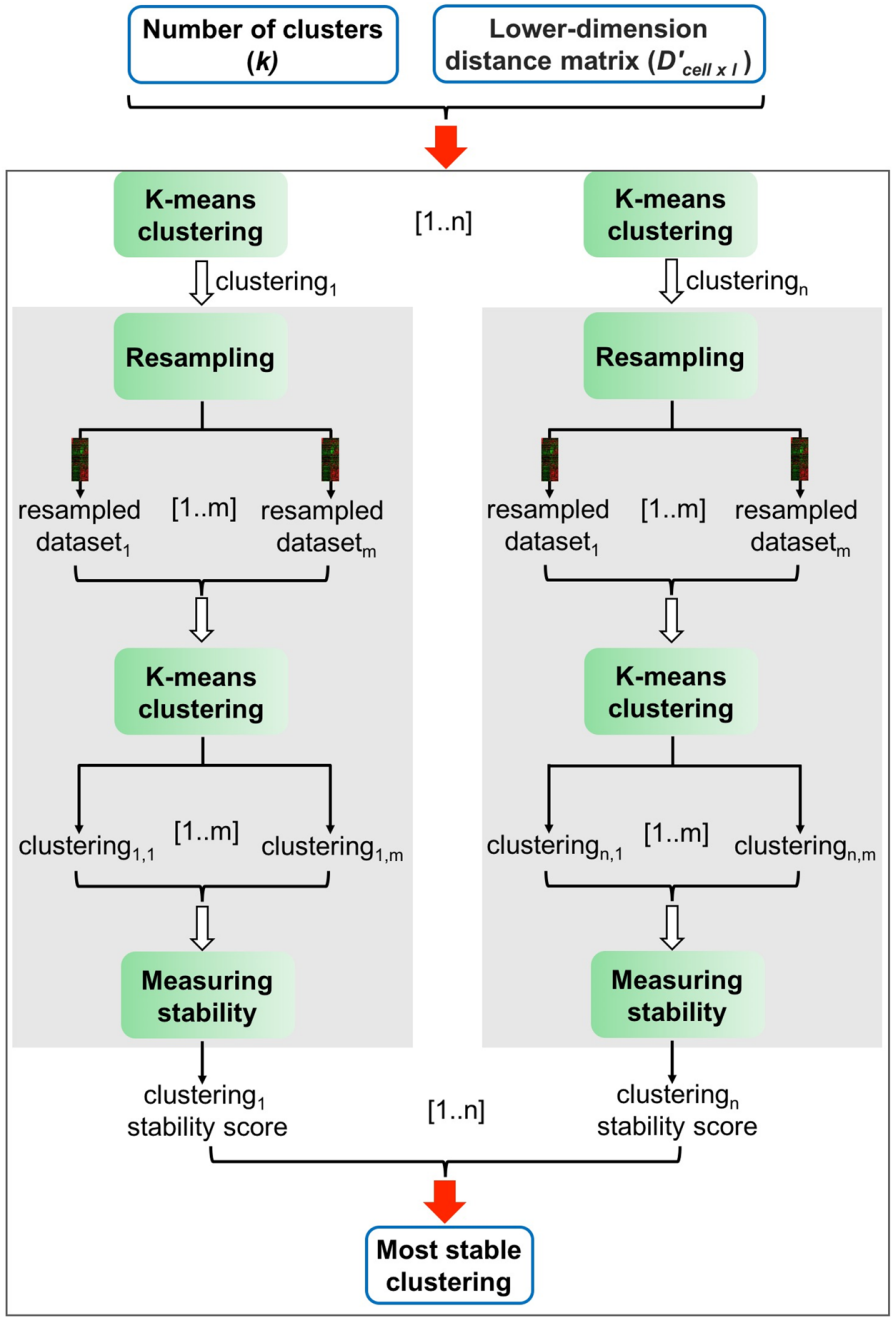
Identifying the most stable clustering. In this analysis, given the lower-dimension distance matrix *D*′_*cell*×*l*_ and the optimal number of clusters *k*, we calculate *n* different clusterings (*clustering*_1_, ..., *clustering*_*n*_) using the k-means clustering algorithm. Then, the stability of each clustering is assessed based on a resampling approach (grey box). A stability score is assigned to each clustering based on how often its clusters are recovered when the input data is perturbed (resampled). A clustering with the maximum stability score is selected as the final solution.

This section describes the detail of the last module of the proposed method. As shown in Fig. 2, using the dimensionality reduced distance matrix *D*′ and the chosen number of clusters *k* from the previous step, we identify the most stable clustering by generating different clustering solutions (*clustering*_*i*_ (*i* ∈ [1..*n*])) and measure the stability of each clustering solution based on a resampling method. The stability measure assigned to each particular clustering (denoted as *clustering*_*i*_) represents how often the *k* clusters belonging to that clustering are preserved when the input data (*D*′) is resampled several times. The resampled datasets are generated from *D*′ by randomly replacing 5% of data points (cells) with noise. These noisy datasets are then used as the input to k-means algorithm. Hence, several clusterings (*clustering*_*i*,*j*_, *j* ∈ [1..*m*]) are generated from the resampled data (resampled versions of *clustering*_*i*_).

In order to assess the stability of each cluster *c* in the *clustering*_*i*_ (original clustering), the cluster *c* is compared to all the clusters in the clustering that is obtained from the resample data (*clustering*_*i*,*j*_) based on the Jaccard distance. *The Jaccard coefficient*^59^*, a similarity measure between sets, is used to compute the similarity between two clusters as follows:*

$${\rm{J}}({\rm{A}},{\rm{B}})=\frac{|A\cap B|}{|A\cup B|},\,A,B\subseteq X$$

where the term A and B are two clusters, consisting of some data points in *X* = {*x*_1_, …, *x*_*N*_}.

If the Jaccard similarity between the cluster *c* (from the original clustering *clustering*_*i*_) and the most similar cluster in the resampled clustering is equal or greater than 0.75, that cluster is considered stable (preserved). Thus, the stability of each cluster in *clustering*_*i*_ is calculated as the percentage of the times that cluster is preserved (Jaccard coefficient ≥ 0.75) across the *m* different resamplings.

We then average the stability measures of the *k* clusters belonging to *clustering*_*i*_, and consider it as the overall stability measure of *clustering*_*i*_. Among *n* different clustering solutions (*clustering*_*i*_ (*i* ∈ [1..*n*])), we select the clustering solution with the maximum stability measure as the final clustering solution.

**Figure 3:**
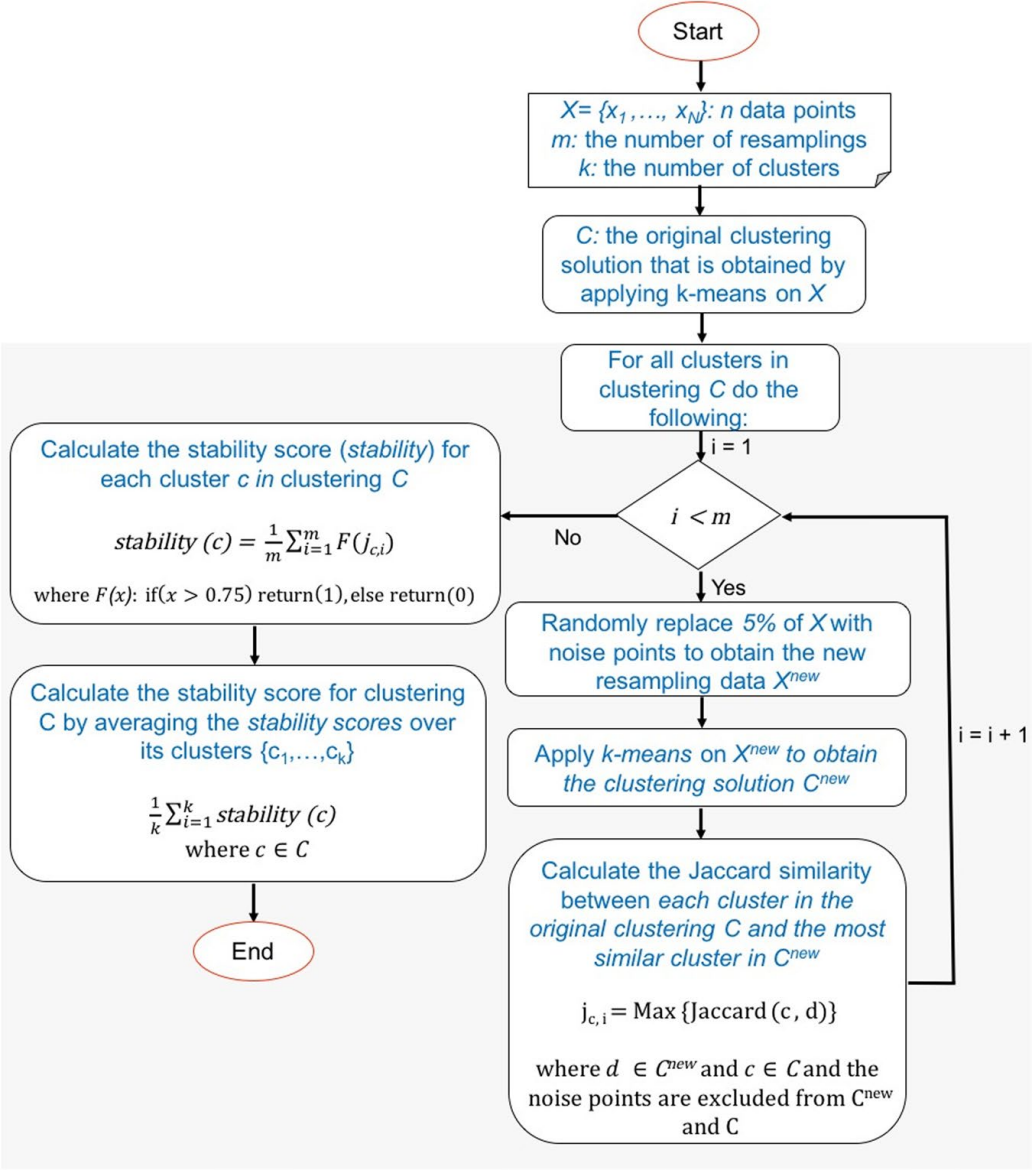
The resampling framework to compute the stability measure for each clustering. The input includes *N* data points *X* = {*x*_1_, ..., *x*_*N*_}, the number of clusters *k*, the number of resamplings *m*, and the clustering *C* that is obtained by applying k-means on *X*. This analysis generates *m* resampling data by randomly replacing 5% of data points with the noise, and computes *m* resampled clusterings based on k-means clustering. Each cluster *c* in *C* is compared with the most similar cluster in the resampling clustering, and the Jaccard coefficient between the two clusters is computed, while the noise points are excluded. The percentage of the times that Jaccard coefficients are larger than 0.75 is considered the stability measure for cluster *c*. The average of stability measures for all clusters belonging to clustering *C* is calculated and considered as the overall stability measure for clustering *C*.

Figure 3 shows the detail of the resampling method we performed to compute the stability measure for each clustering. The clusters that are obtained by applying k-mean on the resampled dataset are compared with the clusters from the original input data only based on the non-noise points (the noise data points are excluded when two clusters are compared based on the Jaccard similarity metric.

### Validation methods

We use 13 different datasets in which the cell types (labels) are known. To measure the level of similarity between the reference labels and the inferred labels that are obtained by each clustering method, we use three different metrics: adjusted rand index (ARI), adjusted mutual information (AMI), and V-measure as explained in the following.

#### Adjusted rand index

Given the cell labels, the Adjusted Rand Index (ARI)^47^ is used to assess the similarity between the inferred clustering and the true clustering. ARI ranges from 0, for poor matching (a random clustering), to 1 for a perfect agreement with the true clustering. For a set of *n* data points, the contingency table is constructed based on the shared number of data points between two clusters. Suppose *X* = {*X*_1_, *X*_2_, ..., *X*_*R*_} and *Y* = {*Y*_1_, *Y*_2_, ..., *Y*_*C*_} represent two different clusterings with R and C clusters, respectively. The overlap between X and Y can be summarized as a contingency table *M*_*R*×*C*_ = [*n*_*ij*_], where *i* = 1...*R*, *j* = 1...*C*. *X*_*i*_ and *Y*_*j*_ denote a cluster in clusterings *X* and *Y*, and i and j refer to the row number and the column number of the contingency table, respectively. The ARI is defined as follow:

1 $$ARI=\frac{\mathop{\sum }\limits_{ij}^{}(\genfrac{}{}{0ex}{}{{n}_{ij}}{2})-[\mathop{\sum }\limits_{i}^{}(\genfrac{}{}{0ex}{}{{a}_{i}}{2})\mathop{\sum }\limits_{j}^{}(\genfrac{}{}{0ex}{}{{b}_{i}}{2})]/(\genfrac{}{}{0ex}{}{n}{2})}{\frac{1}{2}[\mathop{\sum }\limits_{i}^{}(\genfrac{}{}{0ex}{}{{a}_{i}}{2})+\mathop{\sum }\limits_{j}^{}(\genfrac{}{}{0ex}{}{{b}_{i}}{2})]-[\mathop{\sum }\limits_{i}^{}(\genfrac{}{}{0ex}{}{{a}_{i}}{2})\mathop{\sum }\limits_{j}^{}(\genfrac{}{}{0ex}{}{{b}_{i}}{2})]/(\genfrac{}{}{0ex}{}{n}{2})}$$

where *n*_*ij*_ denotes the number of shared data points between clusters *X*_*i*_ and *Y*_*j*_ (*n*_*ij*_ = |*X*_*i*_∩*Y*_*j*_|), and ${a}_{i}={\sum }_{k}{n}_{ik}$ (the sum of the *i*^*th*^ row of the contingency table), and ${b}_{j}={\sum }_{k}{n}_{kj}$ (the sum of the *j*^*th*^ column of the contingency table).

#### Adjusted mutual information

The adjusted mutual information (AMI)^48,49^ is a variation of mutual information that corrects for random partitioning, similar to the way the ARI corrects the rand index. As explained in the previous section, given two different clusterings *X* = {*X*_1_, *X*_2_, ..., *X*_*R*_} and *Y* = {*Y*_1_, *Y*_2_, ..., *Y*_*C*_} of *n* data points with R and C clusters, respectively, the mutual information of cluster overlap between X and Y can be summarized as a contingency table *M*_*R*×*C*_ = [*n*_*ij*_], where *i* = 1...*R*, *j* = 1...*C*, and *n*_*ij*_ represents the number of common data points between clusters *X*_*i*_ and *Y*_*j*_. Suppose a data point is picked at random from X, the probability that the data point falls into cluster *X*_*i*_ is $p(i)=\frac{|{X}_{i}|}{n}$. The entropy^60^ associated with the clustering *X* is calculated as follows:

2 $$H(X)=\mathop{\sum }\limits_{i\mathrm{=1}}^{R}P(i)\,logP(i)$$

*H*(*X*) is non-negative and takes the value 0 only when there is no uncertainty determining a data point's cluster membership (there is only one cluster). The mutual information (*MI*) between two clusterings *X* and *Y* is calculated as follows:

3 $$MI(X,Y)=\mathop{\sum }\limits_{i\mathrm{=1}}^{R}\mathop{\sum }\limits_{j\mathrm{=1}}^{C}P(i,j)\,log\frac{P(i,j)}{P(i)P(j)}$$

where *P*(*i*, *j*) denotes the probability that a data point belongs to both the cluster *X*_*i*_ in *X* and the cluster *Y*_*j*_ in *Y*:

4 $$P(i,j)=\frac{|{X}_{i}\cap {Y}_{j}|}{n}$$

*MI* is a non-negative quantity upper bounded by the entropies H(X) and H(Y). It quantifies the information shared by the two clusterings and therefore can be considered as a clustering similarity measure. The adjusted measure for the mutual information is defined as follows:

5 $$AMI(X,Y)=\frac{MI(X,Y)-E\{MI(X,Y)\}}{max\{H(X),H(Y)\}-E\{MI(X,Y)\}}$$

where the expected mutual information between two random clusterings is:

6 $$E\{MI(X,Y)\}=\mathop{\sum }\limits_{i\mathrm{=1}}^{R}\mathop{\sum }\limits_{j\mathrm{=1}}^{C}\,\mathop{\sum }\limits_{{n}_{ij}=max\mathrm{(1,}{a}_{i}+{b}_{j}-n)}^{min({a}_{i},{b}_{j})}\frac{{n}_{ij}}{n}log\left(\frac{n\mathrm{}.{n}_{ij}}{{a}_{i}{b}_{j}}\right)\frac{{a}_{i}!{b}_{j}!(n-{a}_{i})!(n-{b}_{j})!}{n!{n}_{ij}!({a}_{i}-{n}_{ij})!({b}_{j}-{n}_{ij})!(n-{a}_{i}-{b}_{j}+{n}_{ij})!}$$

where the *a*_*i*_ and *b*_*j*_ are the partial sums of the contingency table: ${a}_{i}={\sum }_{j\mathrm{=1}}^{C}{n}_{ij}$ and ${b}_{j}={\sum }_{i\mathrm{=1}}^{R}{n}_{ij}$.

The adjusted mutual information (*AMI*) takes a value of 1 when the two clusterings are identical and 0 when the *MI* between two partitions equals the value expected due to chance alone.

#### V-measure

The V-measure^50^ is the harmonic mean between two measures: homogeneity and completeness. The homogeneity criteria is satisfied if a clustering assigns *only* those data points that are members of a single class (true cluster) to a single cluster. Thus, the class distribution within each cluster should be skewed to a single class (zero entropy). To determine how close a given clustering is to this ideal, the conditional entropy of the class distribution given the identified clustering is computed as *H*(*C*|*K*), where *C* = {*C*_1_, *C*_2_, ..., *C*_*l*_} is a set of classes and *K* is a clustering *K* = {*K*_1_, *K*_2_, ..., *K*_*m*_}. In the perfectly homogeneous case, this value is 0. However, this value is dependent on the size of the dataset and the distribution of class sizes. Thus, this conditional entropy is normalized by the maximum reduction in entropy the clustering information could provide, *H*(*C*). Therefore, the homogeneity is defined as follows:

7 $$h=\{\begin{array}{cc}1 & \text{if}\,H(C,K)=0\\ 1-\frac{H(C| K)}{H(C)} & \text{otherwise}\end{array}$$

The completeness is symmetrical to homogeneity^50^. In order to satisfy the completeness criteria, a clustering must assign *all* of those data points that are members of a single class to a single cluster. To measure the completeness, the distribution of cluster assignments within each class is assessed. In a perfectly complete clustering solution, each of these distributions will be completely skewed to a single cluster.

Given the homogeneity *h* and completeness *c*, the V-measure is computed as the weighted harmonic mean of homogeneity and completeness:

8 $${\rm{V}} \mbox{-} {\rm{m}}{\rm{e}}{\rm{a}}{\rm{s}}{\rm{u}}{\rm{r}}{\rm{e}}=\frac{(1+\beta )\ast h\ast c}{(\beta \ast h)+c}$$

if *β* is greater than 1, completeness is weighted more strongly in the calculation. If *β* is less than 1, homogeneity is weighted more strongly. Since the computations of homogeneity, completeness and V-measure are completely independent of the number of classes, the number of clusters, the size of the dataset and the clustering algorithm, these measures can be employed for evaluating any clustering solution.

## Results

Tables 1–3 shows the comparison between the proposed method and five other methods: RaceID^35^, SC3^28^, SEURAT^25^, SINCERA^38^, and SNN-Cliq^17^ using the three metrics: ARI, AMI, and V-measures, respectively.

**Table 1:** A comparison between the results of six methods: proposed, RaceID, SC3, Seurat, SINCERA, and SNN-Cliq.

Dataset	#cell types	Proposed		RaceID		SC3		SINCERA		SNN-Cliq		Seurat	
		K (mean ± sd)	ARI (mean ± sd)	K (mean ± sd)	ARI (mean ± sd)	K (mean ± sd)	ARI (mean ± sd)	K	ARI	K	ARI	K	ARI
Biase	3	3 ± 0	**0.94** ± **0.01**	3.14 ± 0.6	0.84 ± 0.25	3 ± 0	**0.94** ± **0**	6	0.71	6	0.66	4	0.78
Deng	10	10 ± 0	0.58 ± 0.02	1 ± 0	0 ± 0	9 ± 0	**0.65** ± **0.002**	3	0.42	17	0.4	6	0.45
Goolam	5	3 ± 0	**0.80** ± **0.09**	1 ± 0	0 ± 0	6 ± 0	0.59 ± 0	13	0.19	17	0.2	3	0.05
Klein	4	6 ± 0	**0.69** ± **0.01**	2.98 ± 0.14	0.48 ± 0.001	19 ± 0	0.44 ± 0.01	43	0.45	265	0.11	3	0
Patel	5	5 ± 0	0.66 ± 0.09	7.44 ± 1.88	0.66 ± 0.08	17 ± 0	0.45 ± 0.01	10	**0.78**	26	0.14	5	0.63
Pollen	11	8 ± 0	0.86 ± 0.02	8.36 ± 2.27	0.55 ± 0.11	10 ± 0	**0.93** ± **0**	10	0.9	22	0.71	8	0.85
Treutlein	5	3 ± 0	**0.72** ± **0.03**	1 ± 0	0 ± 0	3 ± 0	0.66 ± 0	7	0.35	5	0.62	1	0
Yan	8	5 ± 0	**0.81** ± **0.02**	5.5 ± 2.34	0.55 ± 0.17	4 ± 0	0.76 ± 0	8	0.59	13	0.79	3	0.56
sim3	3	3 ± 0	**1** ± **0**	1 ± 0	0 ± 0	3 ± 0	**1** ± **0**	120	0.12	147	0.03	3	**1**
sim4	4	4 ± 0	**0.99** ± **0.005**	1 ± 0	0 ± 0	4 ± 0	**0.99** ± **0.0005**	464	0.08	437	0.01	3	0.57
sim6	6	7.9 ± 0.3	0.56 ± 0.03	1 ± 0	0 ± 0	3 ± 0	0.53 ± 0.005	68	0.25	143	0.06	6	**1**
sim8	8	9.34 ± 0.47	0.77 ± 0.03	1 ± 0	0 ± 0	4 ± 0	0.53 ± 0.04	68	0.35	290	0.05	8	**1**
sim_Tung	8	8 ± 0	**0.42** ± **0**	1 ± 0	0 ± 0	8 ± 0	0 ± 0	17	0.001	77	0.001	8	0

**Figure 4:**
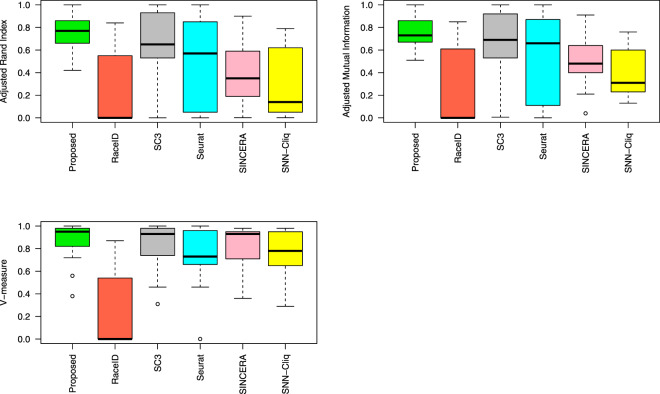
The performance comparison using 13 single cell datasets based on three metrics: the adjusted rand index (ARI), adjusted mutual information (AMI), and V-measure.The proposed method and RaceID were applied 50 times on each dataset. SC3 was used only 5 times on each dataset because it is very stable. The average ARIs, AMIs, and V-measures across different runs are computed for the proposed method, RaceID, and SC3. Since SNN-Cliq, SINCERA, and SEURAT are deterministic, they are run only once for each dataset.

The adjusted rand index (ARI)^47^ is used to evaluate the performance of each clustering method. The proposed method, RaceID, and SC3 are performed 50, 50, and 5 times on each dataset, respectively. SC3 was performed only 5 times because it is very stable (standard deviation of zero for all datasets). The average ARIs across different runs are computed for the proposed method, SC3, and RaceID. Since SNN-Cliq, SINCERA and SEURAT are deterministic, they are performed only once. The proposed method was the best for 8 out of the 13 datasets. The proposed method also yielded the best average ARI, as shown in Fig. 4.

**Table 2:** A comparison between the results of six methods: proposed, RaceID, SC3, Seurat, SINCERA, and SNN-Cliq.

Dataset	#cell types	Proposed		RaceID		SC3		SINCERA		SNN-Cliq		Seurat	
		K (mean ± sd)	AMI (mean ± sd)	K (mean ± sd)	AMI (mean ± sd)	K (mean ± sd)	AMI (mean ± sd)	K	AMI	K	AMI	K	AMI
Biase	3	3 ± 0	**0.92** ± **0.02**	3.14 ± 0.6	0.85 ± 0.23	3 ± 0	**0.92** ± **0**	6	0.64	6	0.62	4	0.74
Deng	10	10 ± 0	0.73 ± 0.01	1 ± 0	0 ± 0	9 ± 0	**0.81** ± **0.006**	3	0.48	17	0.6	6	0.59
Goolam	5	3 ± 0	**0.73** ± **0.04**	1 ± 0	0 ± 0	6 ± 0	0.69 ± 0	13	0.4	17	0.42	3	0.11
Klein	4	6 ± 0	**0.67** ± **0.06**	2.98 ± 0.14	0.51 ± 0.05	19 ± 0	0.53 ± 0.006	43	0.52	265	0.21	3	0.06
Patel	5	5 ± 0	0.86 ± 0.01	7.44 ± 1.88	0.66 ± 0.1	17 ± 0	**0.93** ± **0**	10	0.73	26	0.31	5	0.68
Pollen	11	8 ± 0	0.72 ± 0.01	8.36 ± 2.27	0.68 ± 0	10 ± 0	0.53 ± 0.01	10	**0.91**	22	0.74	8	0.87
Treutlein	5	3 ± 0	0.54 ± 0.03	1 ± 0	0 ± 0	3 ± 0	**0.62** ± **0**	7	0.46	5	0.51	1	0
Yan	8	5 ± 0	**0.78** ± **0.01**	5.5 ± 2.34	0.61 ± 0.17	4 ± 0	0.72 ± 0	8	0.72	13	0.76	3	0.58
sim3	3	3 ± 0	**1** ± **0**	1 ± 0	0 ± 0	3 ± 0	**1** ± **0**	120	0.23	147	0.21	3	**1**
sim4	4	4 ± 0	**0.99** ± **0.007**	1 ± 0	0 ± 0	4 ± 0	**0.99** ± **0.001**	464	0.21	437	0.2	3	0.66
sim6	6	7.9 ± 0.3	0.64 ± 0.02	1 ± 0	0 ± 0	3 ± 0	0.51 ± 0.004	68	0.42	143	0.3	6	**1**
sim8	8	9.34 ± 0.47	0.85 ± 0.01	1 ± 0	0 ± 0	4 ± 0	0.56 ± 0.007	68	0.51	290	0.31	8	**1**
sim_Tung	8	8 ± 0	**0.51** ± **0.008**	1 ± 0	0 ± 0	8 ± 0	0.006 ± 0	17	0.04	77	0.13	8	0

The adjusted mutual information (AMI)^48,49^, is used to evaluate the performance of each clustering method. The proposed method, RaceID, and SC3 are performed 50, 50, and 5 times on each dataset, respectively. The average AMIs across different runs are computed for the proposed method, SC3, and RaceID. Since SNN-Cliq, SINCERA and SEURAT are deterministic, they are performed only once.

**Table 3:** A comparison between the results of six methods: proposed, RaceID, SC3, Seurat, SINCERA, and SNN-Cliq.

Dataset	#cell types	Proposed		RaceID		SC3		SINCERA		SNN-Cliq		Seurat	
		K (mean ± sd)	V-measure (mean ± sd)	K (mean ± sd)	V-measure (mean ± sd)	K (mean ± sd)	V-measure (mean ± sd)	K	V-measure	K	V-measure	K	V-measure
Biase	3	3 ± 0	**0.93** ± **0.03**	3.14 ± 0.6	0.87 ± 0.2	3 ± 0	**0.93** ± **0**	6	0.72	6	0.7	4	0.73
Deng	10	10 ± 0	0.72 ± 0.01	1 ± 0	0 ± 0	9 ± 0	0.74 ± 0.001	3	0.93	17	0.64	6	**0.93**
Goolam	5	3 ± 0	0.82 ± 0.04	1 ± 0	0 ± 0	6 ± 0	**0.98** ± **0**	13	0.71	17	0.65	3	0.66
Klein	4	6 ± 0	0.38 ± 0.01	2.98 ± 0.14	0.4 ± 0.06	19 ± 0	0.31 ± 0.002	43	0.36	265	0.29	3	**0.46**
Patel	5	5 ± 0	0.56 ± 0.02	7.44 ± 1.88	0.54 ± 0.04	17 ± 0	0.46 ± 0.002	10	0.55	26	0.44	5	**0.62**
Pollen	11	8 ± 0	**0.95** ± 0.01	8.36 ± 2.27	0.76 ± 0.03	10 ± 0	0.93 ± 0	10	0.94	22	0.72	8	0.93
Treutlein	5	3 ± 0	**0.96** ± **0**	1 ± 0	0 ± 0	3 ± 0	0.89 ± 0	7	0.93	5	0.92	1	0
Yan	8	5 ± 0	**0.83** ± **0.02**	5.5 ± 2.34	0.68 ± 0.07	4 ± 0	0.81 ± 0	8	0.65	13	0.78	3	0.73
sim3	3	3 ± 0	**1** ± **0**	1 ± 0	0 ± 0	3 ± 0	**1** ± **0**	120	0.95	147	0.95	3	**1**
sim4	4	4 ± 0	**0.99** ± **0.0002**	1 ± 0	0 ± 0	4 ± 0	**0.99** ± **0.00003**	464	0.97	437	0.97	3	0.96
sim6	6	7.9 ± 0.3	0.98 ± 0	1 ± 0	0 ± 0	3 ± 0	0.97 ± 0.0004	68	0.97	143	0.97	6	**1**
sim8	8	9.34 ± 0.47	0.99 ± 0	1 ± 0	0 ± 0	4 ± 0	0.98 ± 0.004	68	0.98	290	0.98	8	**1**
sim_Tung	8	8 ± 0	**0.96** ± **0.03**	1 ± 0	0 ± 0	8 ± 0	0.66 ± 0	17	0.82	77	0.8	8	0.66

The V-measure^50^ is used to evaluate the performance of each clustering method. The proposed method, RaceID, and SC3 are performed 50, 50, and 5 times on each dataset, respectively. The average V-measures across different runs are computed for the proposed method, SC3, and RaceID. Since SNN-Cliq, SINCERA and SEURAT are deterministic, they are performed only once.

We used the R package fpc^61^ to compute the k-means clustering based on the resampling method. We generated 20 different clusterings, and for each clustering we computed 1,000 clusterings based on the resampled datasets to find the most meaningful clustering. We used the log-transformation (*M*′ = *log*2(*M* + 1)) for all methods except SINCERA. For SINCERA we followed the authors instructions^38^ and used the original z-score normalization instead of the log-transformation. In order to generate SC3 results, we used the R package SC3 (http://bioconductor.org/packages/SC3, v.1.8.0). We applied the same gene filtering approach that authors proposed in their study (parameter gene_filter=TRUE).

For SEURAT we used the Seurat R package (v.2.3.4)^62^. We performed the t-SNE using the Rtsne R package with the default parameters, and we used DBSCAN algorithm for clustering. We ran SNN-cliq with the default parameters that are provided by the authors^17^. For RaceID, we used the R code provided by the authors^35^ (https://github.com/dgrun/RaceID).

As shown in Fig. 4, the proposed method performs better than the five methods across 13 different datasets. In this figure, the three boxplots shows the the performance of each method on these 13 datasets based on the adjusted rand index (ARI), adjusted mutual information (AMI), and V-measure. We performed the proposed method, SC3 and RaceID on each dataset for 50, 5, and 50 times, respectively. In these three methods, we calculated the average of ARIs, AMIs, and V-measures over different runs. Since SC3 is reported as a stable method by the authors^28^, we run it only 5 times. Indeed, we have observed the results with a very small standard deviation in all 5 runs for all 13 datasets confirming the claims of the authors. The other clustering methods SEURAT, SINCERA, and SNN-Cliq were run only once since they are deterministic.

## Discussion

The results shown in Tables 1–3 merit some discussion. The Goolam dataset, for instance, includes 5 true cell types. On this dataset, the proposed algorithm identifies 3 clusters, while SC3 identifies 6, RaceID 1, Seurat 2, SINCERA 13 and SNN-Cliq 17 types. Even though the number of clusters closest to the number of true types is 6, as yielded by SC3, the membership of various cells in these clusters is not correct since the ARI index associated to these 6 clusters is only 0.59 compared to the ARI index of 0.8 associated to the 3 clusters constructed by the proposed method.

Conversely, for the Patel dataset that includes 5 cell types, the proposed method was able to correctly estimate the number of clusters (k = 5). However, the distribution of the individual cells across these five clusters is not perfect, as illustrated by the lower ARI value of 0.66, compared to the 0.78 ARI associated with the SINCERA results.

As another observation, the Pollen dataset includes 11 cell types. Using this dataset, the number of clusters (k = 10) determined by SINCERA is close to the correct number of cell types. However, SC3 achieved better clustering (ARI = 0.93) in contrast to the five other methods. SC3 identified 17 different clusters using this dataset.

Two conclusions may be drawn from these observations. First, results should not be assessed based on the agreement between the number of clusters found and the number of known cell types – the assignment of each cell to a given type is more important. Second, larger number of clusters reported will be associated with larger values of ARI. Therefore, results that include very large number of clusters should be regarded with caution.

RaceID and Seurat both were not able to find a meaningful clustering for the Treutlein dataset. The identified number of clusters by both RaceID and Seurat is 1 (k = 1), while this dataset includes 5 different cell types. As a result, the clusterings obtained by these two methods are poorly matched to the reference clustering. In Deng dataset, the best ARI of 0.65 is obtained by SC3 but this value is not very high. The poor results obtained by all 6 methods using this dataset might be due to noisy data.

We also assessed the reproducibility/stability of the stochastic methods: proposed, RaceID, and SC3 by running each method several times. Although SC3’s consensus pipeline provides a very stable solution (very low standard deviation for the three metrics and *k* across all datasets), it is computationally more costly than other methods. In summary, one key advantage of our proposed method is that we produce consistent clustering across different datasets.

**Figure 5:**
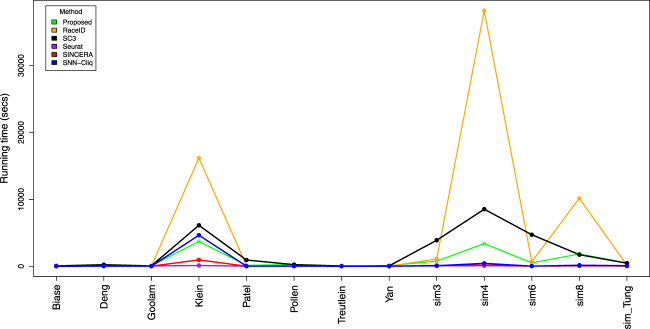
The run time of the different methods using 13 single cell datasets.

The run time for each method using 13 different datasets is shown in Fig. 5. It is notable that RaceID, the proposed method, and SC3 have a non-linear increase in run time. At this time, it appears that it is unfeasible to perform this method on large datasets consisting of thousands of cells. The fastest method among all the methods is Seurat, which is a graph-based method. The graph-based methods often return only a single clustering solution with a faster run time and they do not require the user to provide the number of clusters^33^. Seurat is a popular choice for the large data sets based on the its optimal speed and scalability. However, it has been shown that Seurat does not provide an accurate solution for smaller datasets^33^. The details of the run times are included in Supplementary Materials.

More generally, finding an optimal clustering method that provides stable solutions for all situations may not be possible. In fact, because no method can perform well for all situations, a comparative analysis of methods based on a set of criteria should be employed^33^.

## Conclusion

Recent advances in single-cell RNA-Seq (scRNASeq) provide the opportunity to perform single-cell transcriptome analysis. In this paper, we develop a pipeline to cluster the individual cells based on their gene expression values such that each cluster consisting of cells with specific functions or distinct developmental stages. We first filter genes that are not expressed in any cell. Then, we compute the distance between the cells using the Euclidean distance. We reduce the dimensions of the distance matrix data using the t-distributed stochastic neighbor embedding (t-SNE) technique. Based on the dimensionality reduced distance matrix, we explore strong patterns (clusters) of cells by randomly drawing a percentage of the data points without replacement, and replacing them with points from a noise distribution. We apply the proposed method on 13 different single cell datasets, and we compare it with five related methods: RaceID, SC3, Seurat, SINCERA, and SNN-Cliq. The results of the evaluation on datasets demonstrate that the proposed method yields better clustering results in comparison to the existing methods.

## Supplementary information

Supplementary information.
