# Supplementary material for: Identification of cell types from single cell data using stable clustering
Source: Sci Rep. 2020 Jul 23;10:12349. doi: 10.1038/s41598-020-66848-3 (PMC7378075; doi:10.1038/s41598-020-66848-3)
Supplement: Supplementary file 1 — Supplementary information. [file 41598_2020_66848_MOESM1_ESM.pdf]

Identification of cell types from single cell data using stable clustering

Supplementary Materials

Azam Peyvandipour[1], Adib Shafi[1], Nafiseh Saberian[1], and Sorin Draghici[1,2*]

[1]Department of Computer Science, Wayne State University, Detroit, MI 48202

[2]Department of Obstetrics and Gynecology, Wayne State University, Detroit, MI 48202

Contents

1 Data source **2**

2 The run time of the six different methods **3**

3 Uniform manifold approximation and projection (UMAP) for the dimension reduction **3**

4 Resampling-based k-means clustering **7**

1 Data source

The list of eight single cell datasets we used in our analysis is summarized in Table 1.

We simulated four scRNA-seq datasets with varying degree of cluster separability using the splatter R package [19].The dataset *sim*3, consists of 3 subpopulations (1000 cells) with relative abundances 0.35, 0.30, and 0.35. The dataset *sim*4 includes 4 subpopulations (3000 cells) with relative abundances 0.15, 0.3, 0.2, and 0.35. The dataset *sim*6 includes 6 subpopulations (1000 cells) with relative abundances 0.3, 0.1,0.1, 0.2, 0.2, and 0.1. Finally, the dataset *sim*8 consists of 8 subpopulations (2000 cells) with relative abundances 0.05, 0.1,0.1, 0.2, 0.2, 0.1, 0.15, and 0.1. We also used SPARSim R package [1] to generate a simulation dataset with 8 subpopulations (564 cells) from the real dataset Tung [15].

Table 1: **Single cell datasets**. All the datasets, except Klein, Patel, and Treutlein are considered as "gold standard". In the "gold standard" datasets, the cell types are clearly known. Klein, Patel, and Treutlein are referred as "silver standard' by [7] since the cell types are determined based on the computational methods and the authors' knowledge of the underlying biology.

Dataset	# cell type	Organism	#cell	Source	Reference
Biase	3	Mouse	49	Embryo development	[2]
Deng	10	Mouse	268	Embryo development	[3]
Goolam	5	Mouse	124	Embryo development	[4]
Klein	4	Mouse	2717	Embryo Stem Cells	[8]
Patel	5	Human	430	Tissues	[11]
Pollen	11	Human	301	Tissues	[12]
Treutlein	5	Mouse	80	Tissues	[14]
Yan	8	Human	124	Embryo development	[18]

2 The run time of the six different methods

The run time for each method using 8 different datasets is shown in Table 2.

Table 2: The run time (secs) of the different methods using 13 single cell datasets, including 5 simulation datasets.

	Proposed	RaceID	SC3	Seurat	SINCERA	SNN-Cliq
Biase	43.47	8.79	59.79	27.3	3.56	0.62
Deng	188.51	55.37	234.3	23.68	10	16.39
Goolam	86.55	15.69	59.52	41.36	14.16	5.75
Klein	3709.28	16175.58	6127.56	117.1	952.25	4643.8
patel	154.18	100.32	933.05	13.78	10.25	12.22
Pollen	257.14	50.5	245.02	25.86	13.11	17.35
treutlein	53.49	8.34	47.89	23.09	5.82	1.33
Yan	87.74	13.46	60.31	19.5	4.96	2.23
sim3	777.08	1102.2	3888	63	118.8	84.71
sim4	3381.1	38232	8532	85.2	170.4	439.12
sim6	510.48	670.2	4716	31.09	18.3	23.01
sim8	1839.75	10160.64	1733.4	53.33	85.2	157.47
sim_Tung	504.6	171.6	458.4	40.66	37.36	86.13

3 Uniform manifold approximation and projection (UMAP) for the dimension reduction

We applied the Uniform Manifold Approximation and Projection (UMAP) [10] to reduce the dimensionality of the distance matrix in which the rows and columns are the cells. Then, we identified the most stable clustering of cells using the lower-dimension distance matrix based on the resampled-based k-means clustering. As shown in Figure 1, we found that the performance is better when t-SNE is used for the dimensionality reduction.

Tables 3–5 show the comparison between the results of six methods: K-means-UMAP, RaceID, SC3, Seurat, SINCERA, and SNN-Cliq .The K-means-UMAP is an alternative to the proposed method in which UMAP technique is employed for the dimensionality reduction.

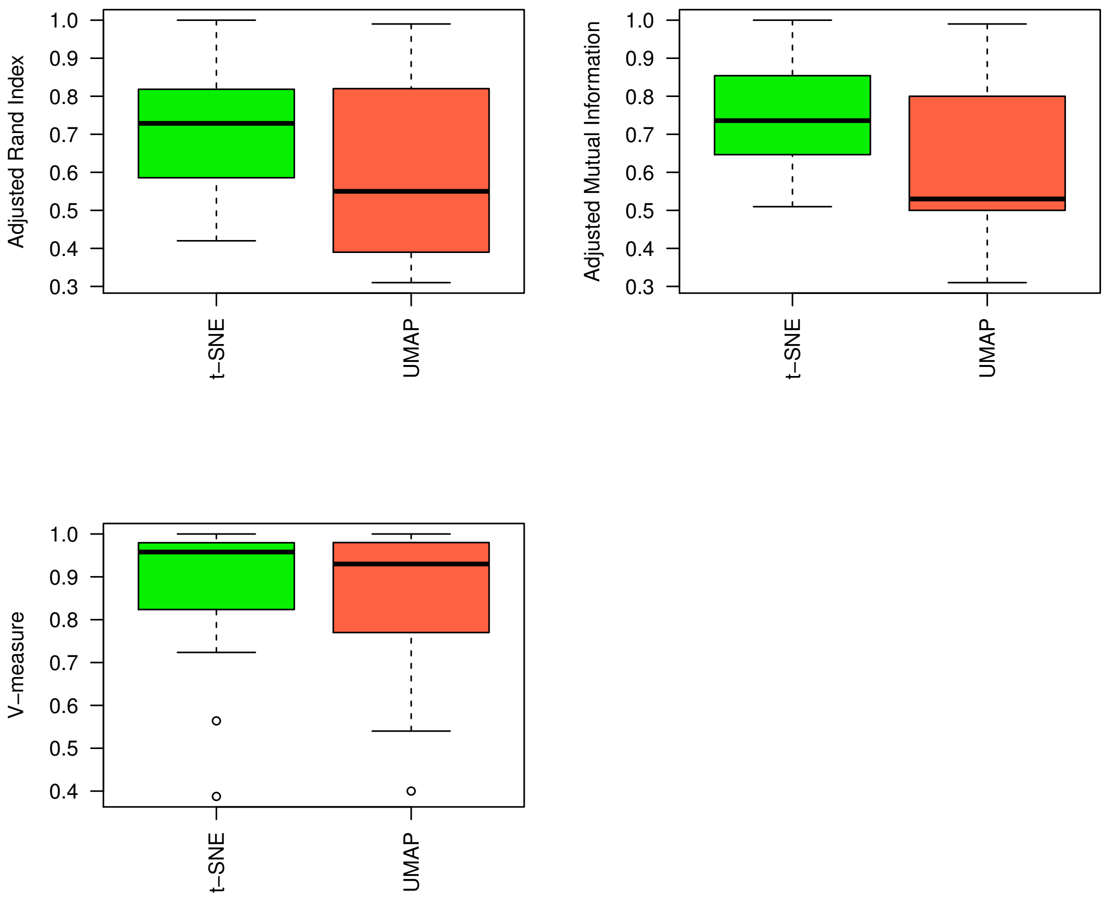

Figure 1: **The comparison between the results of the resampling-based k-means clustering method based on two different dimensionality reduction techniques: the t-distributed stochastic neighbor embedding (t-SNE) [9] and Uniform Manifold Approximation and Projection (UMAP) [10]**. In this comparison, 13 datasets, including 8 real single cell gene expression datasets are used. The results are compared based on the three metrics: the adjusted rand index (ARI), adjusted mutual information (AMI), and V-measure. The results show that the t-SNE dimensionality reduction technique provides better performance in comparison to the UMAP technique.

Dataset	#cell types	K-means-UMAP		RaceID		SC3		SINCERA		SNN-Cliq		Seurat	
		K (mean±sd)	ARI (mean±sd)	K (mean±sd)	ARI (mean±sd)	K (mean±sd)	ARI (mean±sd)	K	ARI	K	ARI	K	ARI
Biase	3	3±0	0.95±0	3.14±0.6	0.84±0.25	3±0	0.94±0	6	0.71	6	0.66	4	0.78
Deng	10	5±0	0.39±0.02	1±0	0±0	9±0	0.65±0.002	3	0.42	17	0.4	6	0.45
Goolam	5	2.88±0.33	0.82±0.11	1±0	0±0	6±0	0.59±0	13	0.19	17	0.2	3	0.05
Klein	4	3±0	0.55±0	2.98±0.14	0.48±0.001	19±0	0.44±0.01	43	0.45	265	0.11	3	0
Patel	5	2.76±0.52	0.31±0.07	7.44±1.88	0.66±0.08	17±0	0.45±0.01	10	0.78	26	0.14	5	0.63
Pollen	11	7.98±0.14	0.84±0.03	8.36±2.27	0.55±0.11	10±0	0.93±0	10	0.9	22	0.71	8	0.85
Treutlein	5	3.24±0.43	0.51±0.12	1±0	0±0	3±0	0.66±0	7	0.35	5	0.62	1	0
Yan	8	8±0	0.75±0.1	5.5±2.34	0.55±0.17	4±0	0.76±0	8	0.59	13	0.79	3	0.56
sim3	3	3±0	0.99±0.01	1±0	0±0	3±0	1±0	120	0.12	147	0.03	3	1
sim4	4	3±0	0.55±0	1±0	0±0	4±0	0.99±0.0005	464	0.08	437	0.01	3	0.57
sim6	6	16.72±0.45	0.32±0.02	1±0	0±0	3±0	0.53±0.005	68	0.25	143	0.06	6	1
sim8	8	18±0	0.38±0.02	1±0	0±0	4±0	0.53±0.04	68	0.35	290	0.05	8	1
sim_Tung	8	8±0	0.41±0.01	1±0	0±0	8±0	0±0	17	0.001	77	0.001	8	0

Table 3: **A comparison between the results of six methods: K-means-UMAP, RaceID, SC3, Seurat, SINCERA, and SNN-Cliq.** The K-means-UMAP is an alternative to the proposed method in which UMAP technique is employed for the dimensionality reduction. The adjusted rand index (ARI) [6] is used to evaluate the performance of each clustering method. The K-means-UMAP method, RaceID, and SC3 are performed 50, 50, and 5 times on each dataset, respectively. SC3 was performed only 5 times because it is very stable (standard deviation of zero for all datasets). The average ARIs across different runs are computed for the K-means-UMAP, SC3, and RaceID. Since SNN-Cliq, SINCERA and SEURAT are deterministic, they are performed only once. For each dataset, the best ARI is highlighted in green. The alternative approaches:

Dataset	#cell types	K-means-UMAP		RaceID		SC3		SINCERA		SNN-Cliq		Seurat	
		K (mean±sd)	AMI (mean±sd)	K (mean±sd)	AMI (mean±sd)	K (mean±sd)	AMI (mean±sd)	K	AMI	K	AMI	K	AMI
Biase	3	3±0	0.92±0	3.14±0.6	0.85±0.23	3±0	0.92±0	6	0.64	6	0.62	4	0.74
Deng	10	5±0	0.51±0.01	1±0	0±0	9±0	0.81±0.006	3	0.48	17	0.6	6	0.59
Goolam	5	2.88±0.33	0.72±0.1	1±0	0±0	6±0	0.69±0	13	0.4	17	0.42	3	0.11
Klein	4	3±0	0.53±0	2.98±0.14	0.51±0.05	19±0	0.53±0.006	43	0.52	265	0.21	3	0.06
Patel	5	2.76±0.52	0.31±0.07	7.44±1.88	0.66±0.1	17±0	0.93±0	10	0.73	26	0.31	5	0.68
Pollen	11	7.98±0.14	0.85±0.02	8.36±2.27	0.68±0	10±0	0.53±0.01	10	0.91	22	0.74	8	0.87
Treutlein	5	3.24±0.43	0.44±0.06	1±0	0±0	3±0	0.62±0	7	0.46	5	0.51	1	0
Yan	8	8±0	0.80±0.04	5.5±2.34	0.61±0.17	4±0	0.72±0	8	0.72	13	0.76	3	0.58
sim3	3	3±0	0.99±0.02	1±0	0±0	3±0	1±0	120	0.23	147	0.21	3	1
sim4	4	4±0	0.61±0	1±0	0±0	4±0	0.99±0.001	464	0.21	437	0.2	3	0.66
sim6	6	16.72±0.45	0.48±0.01	1±0	0±0	3±0	0.51±0.004	68	0.42	143	0.3	6	1
sim8	8	18±0	0.53±0.01	1±0	0±0	4±0	0.56±0.007	68	0.51	290	0.31	8	1
sim_Tung	8	8±0	0.50±0.01	1±0	0±0	8±0	0.006±0	17	0.04	77	0.13	8	0

Table 4: **A comparison between the results of six methods: K-means-UMAP, RaceID, SC3, Seurat, SINCERA, and SNN-Cliq.** The K-means-UMAP is an alternative to the proposed method in which UMAP technique is employed for the dimensionality reduction. The adjusted mutual information (AMI) [16, 17], is used to evaluate the performance of each clustering method. The K-means-UMAP, RaceID, and SC3 are performed 50, 50, and 5 times on each dataset, respectively. The average AMIs across different runs are computed for the K-means-UMAP, SC3, and RaceID. Since SNN-Cliq, SINCERA and SEURAT are deterministic, they are performed only once.

Dataset	#cell types	K-means-UMAP		RaceID		SC3		SINCERA		SNN-Cliq		Seurat	
		K (mean±sd)	V-measure (mean±sd)	K (mean±sd)	V-measure (mean±sd)	K (mean±sd)	V-measure (mean±sd)	K	V-measure	K	V-measure	K	V-measure
Biase	3	3±0	0.93±0	3.14±0.6	0.87±0.2	3	0.93±0	6	0.72	6	0.7	4	0.73
Deng	10	5±0	0.75±0.06	1±0	0±0	9	0.74±0.001	3	0.93	17	0.64	6	0.93
Goolam	5	2.88±0.33	0.84±0.04	1±0	0±0	6	0.98±0	13	0.71	17	0.65	3	0.66
Klein	4	3±0	0.40±0	2.98±0.14	0.4±0.06	19	0.31±0.002	43	0.36	265	0.29	3	0.46
Patel	5	2.76±0.52	0.54±0.02	7.44±1.88	0.54±0.04	17	0.46±0.002	10	0.55	26	0.44	5	0.62
Pollen	11	7.98±0.14	0.94±0.02	8.36±2.27	0.76±0.03	10	0.93±0	10	0.94	22	0.72	8	0.93
Treutlein	5	3.24±0.43	0.94±0.01	1±0	0±0	3	0.89±0	7	0.93	5	0.92	1	0
Yan	8	8±0	0.77±0.07	5.5±2.34	0.68±0.07	4	0.81±0	8	0.65	13	0.78	3	0.73
sim3	3	3±0	1±0	1±0	0±0	3	1±0	120	0.95	147	0.95	3	1
sim4	4	3±0	0.98±0	1±0	0±0	4	0.99±0.00003	464	0.97	437	0.97	3	0.96
sim6	6	16.72±0.45	0.98±0	1±0	0±0	3	0.97±0.0004	68	0.97	143	0.97	6	1
sim8	8	18±0	0.99±0	1±0	0±0	4	0.98±0.004	68	0.98	290	0.98	8	1
sim_Tung	8	8±0	0.91±0.01	1±0	0±0	8	0.66±0	17	0.82	77	0.80	8	0.66

Table 5: **A comparison between the results of six methods: K-means-UMAP, RaceID, SC3, Seurat, SINCERA, and SNN-Cliq.** The K-means-UMAP is an alternative to the proposed method in which UMAP technique is employed for the dimensionality reduction. The K-means-UMAP, RaceID, and SC3 are performed 50, 50, and 5 times on each dataset, respectively. The average V-measures across different runs are computed for the K-means-UMAP, SC3, and RaceID. Since SNN-Cliq, SINCERA and SEURAT are deterministic, they are performed only once. For each dataset, the best V-measure is highlighted in green.

4 Resampling-based k-means clustering

We replaced the 5% of the data points with noise (noise tuning threshold=0.05). In [5], Hennig performed a comparative analysis using two thresholds 0.05 and 0.2 and showed that the threshold 0.05 provides more stable clusterings. We assessed the performance of our proposed method using other noise tuning thresholds: 0.1 and 0.2. Tables 6–8 show comparison between the proposed method (using 3 thresholds) and five other methods based on the three metrics: the adjusted rand index (ARI), adjusted mutual information (AMI), and V-measure. Indeed, we have observed the threshold 0.05 provides better performance.

To assess the stability of a cluster, we used the same threshold (0.75) that is recommended by Hennig [5]. In this study [5], it has been shown that a stable cluster will yield a Jaccard similarity value of 0.75 or more. Thus, if the Jaccard similarity between the cluster (from original clustering) and the most similar cluster in the resampled clustering is equal or greater than 0.75, that the cluster is considered as successfully recovered.

Dataset	#cell types	Proposed- ARI (mean±sd)			RaceID	SC3	SINCERA	SNN-Cliq	Seurat
		Threshold=0.05	Threshold=0.1	Threshold=0.2	ARI (mean±sd)	ARI (mean±sd)	ARI	ARI	ARI
Biase	3	0.94±0.01	0.95±0.01	0.91±0.15	0.84±0.25	0.94±0	0.71	0.66	0.78
Deng	10	0.58±0.02	0.43±0.03	0.43±0.01	9±0	0.65±0.002	0.42	0.4	0.45
Goolam	5	0.80±0.09	0.75±0.12	0.69±0.13	0±0	0.59±0	0.19	0.20	0.05
Klein	4	0.69±0.01	0.70±0.01	0.70±0.03	0.48±0.001	0.44±0.01	0.45	0.11	0
Patel	5	0.66±0.09	0.96±0.01	0.95±0.01	0.66±0.08	0.45±0.01	0.78	0.14	0.63
Pollen	11	0.86±0.02	0.89±0.04	0.85±0.05	0.55±0.11	0.93±0	0.90	0.71	0.85
Treutlein	5	0.72±0.03	0.32±0.04	0.31±0.04	0±0	0.66±0	0.35	0.62	0
Yan	8	0.81±0.02	0.82±0.09	0.90±0.03	0.55±0.17	0.76±0	0.59	0.79	0.56
sim3	3	1±0	1±0	1±0	0±0	1±0	0.12	0.03	1
sim4	4	0.99±0.005	0.90±0.08	0.89±0.08	0±0	0.99±0.0005	0.08	0.01	0.57
sim6	6	0.56±0.03	0.59±0.03	0.56±0.05	0±0	0.53±0.005	0.25	0.06	1
sim8	8	0.77±0.03	0.78±0.04	0.79±0.045	0±0	0.53±0.04	0.35	0.05	1
sim_Tung	8	0.42±0	0±0	0±0	0±0	0±0	0.001	0.001	0

Table 6: **A comparison between the results of six methods: proposed, RaceID, SC3, Seurat, SINCERA, and SNN-Cliq.** The adjusted rand index (ARI) [6] is used to evaluate the performance of each clustering method. The proposed method was performed based on three noise tuning thresholds: 0.05, 0.1 and 0.2. The proposed method, RaceID, and SC3 are performed 50, 50, and 5 times on each dataset, respectively. SC3 was performed only 5 times because it is very stable (standard deviation of zero for all datasets). The average ARIs across different runs are computed for the proposed method, SC3, and RaceID. Since SNN-Cliq, SINCERA and SEURAT are deterministic, they are performed only once. For each dataset, the best ARI is highlighted in green.

Dataset	#cell types	Proposed- AMI (mean±sd)			RaceID	SC3	SINCERA	SNN-Cliq	Seurat
		Threshold=0.05	Threshold=0.1	Threshold=0.2	AMI (mean±sd)	AMI (mean±sd)	AMI	AMI	AMI
Biase	3	0.92±0.02	0.88±0.15	0.90±0.07	0.85±0.23	0.92±0	0.64	0.62	0.74
Deng	10	0.73±0.01	0.58±0.03	0.58±0.02	0±0	0.81±0.006	0.48	0.6	0.59
Goolam	5	0.73±0.04	0.73±0.05	0.71±0.05	0±0	0.69±0	0.4	0.42	0.11
Klein	4	0.67±0.06	0.73±0.01	0.73±0.02	0.51±0.05	0.53±0.006	0.52	0.21	0.06
Patel	5	0.86±0.01	0.94±0.03	0.94±0.01	0.66±0.1	0.93±0 0	0.73	0.31	0.68
Pollen	11	0.72±0.01	0.89±0.02	0.88±0.02	0.68±0	0.53±0.01	0.91	0.74	0.87
Treutlein	5	0.54±0.03	0.41±0.04	0.42±0.04	0±0	0.62±0	0.46	0.51	0
Yan	8	0.78±0.01	0.78±0.01	0.89±0.04	0.61±0.17	0.72±0	0.72	0.76	0.58
sim3	3	1±0	1±0	1±0.01	0±0	1±0	0.23	0.21	1
sim4	4	0.99±0.007	0.91±0.07	0.89±0.07	0±0	0.99±0.001	0.21	0.2	0.66
sim6	6	0.64±0.02	0.66±0.02	0.65±0.03	0±0	0.51±0.004	0.42	0.3	1
sim8	8	0.85±0.01	0.85±0.02	0.85±0.02	0±0	0.56±0.007	0.51	0.31	1
sim_Tung	8	0.51±0.008	0.01±0	0.01±0	0±0	0.006±0	0.04	0.13	0

Table 7: **A comparison between the results of six methods: proposed, RaceID, SC3, Seurat, SINCERA, and SNN-Cliq.**The adjusted mutual information (AMI) [16, 17], is used to evaluate the performance of each clustering method. The proposed method was performed based on three noise tuning thresholds: 0.05, 0.1 and 0.2. The proposed method, RaceID, and SC3 are performed 50, 50, and 5 times on each dataset, respectively. The average AMIs across different runs are computed for the proposed method, SC3, and RaceID. Since SNN-Cliq, SINCERA and SEURAT are deterministic, they are performed only once. For each dataset, the best AMI is highlighted in green.

Dataset	#cell types	Proposed- V-measure (mean±sd)			RaceID	SC3	SINCERA	SNN-Cliq	Seurat
		Threshold=0.05	Threshold=0.1	Threshold=0.2	V-measure (mean±sd)	V-measure (mean±sd)	V-measure	V-measure	V-measure
Biase	3	0.93±0.03	0.93±0.02	0.90±0.1	0.87±0.2	0.93±0	0.72 6	0.7	0.73
Deng	10	0.72±0.01	0.75±0.06	0.81±0.1	0±0	0.74±0.001	0.93	0.64	0.93
Goolam	5	0.82±0.04	0.86±0.09	0.85±0.09	0±0	0.98±0	0.71	0.65	0.66
Klein	4	0.38±0.01	0.39±0.01	0.40±0.03	0.40±0.02	0.31±0.002	0.36	0.29	0.46
Patel	5	0.56±0.02	0.81±0.03	0.81±0.03	0.54±0.04	0.46±0.002	0.55	0.44	0.62
Pollen	11	0.95±0.01	0.92±0.02	0.91±0.02	0.76±0.03	0.93±0	0.94	0.72	0.93
Treutlein	5	0.96±0	0.93±0.01	0.93±0.01	0±0	0.89±0	0.93	0.92	0
Yan	8	0.83±0.02	0.85±0.04	0.87±0.04	0.68±0.07	0.81±0	0.65	0.78	0.73
sim3	3	1±0	1±0	1±0	0±0	1±0	0.95	0.95	1
sim4	4	0.99±0.0002	0.99±0.003	0.99±0	0±0	0.99±0.00003	0.97	0.97	0.96
sim6	6	0.98±0	0.99±0.001	0.99±0	0.99±0	0.99±0.0004	0.97	0.97	1
sim8	8	0.99±0	0.99±0.0009	1±0	0±0	0.98±0.004	0.98	0.98	1
sim_Tung	8	0.96±0.03	0.71±0.01	0.71±0.01	0±0	0.66±0	0.82	0.80	0.66

Table 8: **A comparison between the results of six methods: proposed, RaceID, SC3, Seurat, SINCERA, and SNN-Cliq.** The V-measure [13] is used to evaluate the performance of each clustering method. The proposed method was performed based on three noise tuning thresholds: 0.05, 0.1 and 0.2. The proposed method, RaceID, and SC3 are performed 50, 50, and 5 times on each dataset, respectively. The average V-measures across different runs are computed for the proposed method, SC3, and RaceID. Since SNN-Cliq, SINCERA and SEURAT are deterministic, they are performed only once. For each dataset, the best V-measure is highlighted in green.

References

[1] Giacomo Baruzzo, Ilaria Patuzzi, and Barbara Di Camillo. Sparsim single cell: a count data simulator for scrna-seq data. *Bioinformatics*, 2019.

[2] Fernando H Biase, Xiaoyi Cao, and Sheng Zhong. Cell fate inclination within 2-cell and 4-cell mouse embryos revealed by single-cell RNA sequencing. *Genome Research*, 24(11):1787–1796, 2014.

[3] Qiaolin Deng, Daniel Ramsköld, Björn Reinius, and Rickard Sandberg. Single-cell RNA-seq reveals dynamic, random monoallelic gene expression in mammalian cells. *Science*, 343(6167):193–196, 2014.

[4] Mubeen Goolam, Antonio Scialdone, Sarah JL Graham, Iain C Macaulay, Agnieszka Jedrusik, Anna Hupalowska, Thierry Voet, John C Marioni, and Magdalena Zernicka-Goetz. Heterogeneity in Oct4 and Sox2 targets biases cell fate in 4-cell mouse embryos. *Cell*, 165(1):61–74, 2016.

[5] Christian Hennig. Cluster-wise assessment of cluster stability. *Computational Statistics & Data Analysis*, 52(1):258–271, 2007.

[6] Lawrence Hubert and Phipps Arabie. Comparing partitions. *Journal of Classification*, 2(1):193–218, 1985.

[7] Vladimir Yu Kiselev, Kristina Kirschner, Michael T Schaub, Tallulah Andrews, Andrew Yiu, Tamir Chandra, Kedar N Natarajan, Wolf Reik, Mauricio Barahona, Anthony R Green, et al. SC3: consensus clustering of single-cell RNA-seq data. *Nature Methods*, 14(5):483, 2017.

[8] Allon M Klein, Linas Mazutis, Ilke Akartuna, Naren Tallapragada, Adrian Veres, Victor Li, Leonid Peshkin, David A Weitz, and Marc W Kirschner. Droplet barcoding for single-cell transcriptomics applied to embryonic stem cells. *Cell*, 161(5):1187–1201, 2015.

[9] Laurens van der Maaten and Geoffrey Hinton. Visualizing data using t-SNE. *Journal of Machine Learning Research*, 9(Nov):2579–2605, 2008.

[10] Leland McInnes, John Healy, and James Melville. Umap: Uniform manifold approximation and projection for dimension reduction. *arXiv preprint arXiv:1802.03426*, 2018.

[11] Anoop P. Patel, Itay Tirosh, John J. Trombetta, Alex K. Shalek, Shawn M. Gillespie, Hiroaki Wakimoto, Daniel P. Cahill, Brian V. Nahed, William T. Curry, Robert L. Martuza, David N. Louis, Orit Rozenblatt-Rosen, Mario L. Suvà, Aviv Regev, and Bradley E. Bernstein. Single-cell RNA-seq highlights intratumoral heterogeneity in primary glioblastoma. *Science*, 344(6190):1396–1401, June 2014.

[12] Alex A. Pollen, Tomasz J. Nowakowski, Joe Shuga, Xiaohui Wang, Anne A. Leyrat, Jan H. Lui, Nianzhen Li, Lukasz Szpankowski, Brian Fowler, Peilin Chen, Naveen Ramalingam, Gang Sun, Myo Thu, Michael Norris, Ronald Lebofsky, Dominique Toppani, Darnell W. Kemp Ii, Michael Wong, Barry Clerkson, Brittnee N. Jones, Shiquan Wu, Lawrence Knutsson, Beatriz Alvarado, Jing Wang, Lesley S. Weaver, Andrew P. May, Robert C. Jones, Marc A. Unger, Arnold R. Kriegstein, and Jay A. A. West. Low-coverage single-cell mRNA sequencing reveals cellular heterogeneity and activated signaling pathways in developing cerebral cortex. *Nature Biotechnology*, 32(10):1053–1058, October 2014.

[13] Andrew Rosenberg and Julia Hirschberg. V-measure: A conditional entropy-based external cluster evaluation measure. In *Proceedings of the 2007 joint conference on empirical methods in natural language processing and computational natural language learning (EMNLP-CoNLL)*, pages 410–420, 2007.

[14] Barbara Treutlein, Doug G Brownfield, Angela R Wu, Norma F Neff, Gary L Mantalas, F Hernan Espinoza, Tushar J Desai, Mark A Krasnow, and Stephen R Quake. Reconstructing lineage hierarchies of the distal lung epithelium using single-cell RNA-seq. *Nature*, 509(7500):371, 2014.

[15] Po-Yuan Tung, John D Blischak, Chiaowen Joyce Hsiao, David A Knowles, Jonathan E Burnett, Jonathan K Pritchard, and Yoav Gilad. Batch effects and the effective design of single-cell gene expression studies. *Scientific Reports*, 7:39921, 2017.

[16] Nguyen Xuan Vinh, Julien Epps, and James Bailey. Information theoretic measures for clusterings comparison: is a correction for chance necessary? In *Proceedings of the 26th annual international conference on machine learning*, pages 1073–1080, 2009.

[17] Nguyen Xuan Vinh, Julien Epps, and James Bailey. Information theoretic measures for clusterings comparison: Variants, properties, normalization and correction for chance. *Journal of Machine Learning Research*, 11(Oct):2837–2854, 2010.

[18] Liying Yan, Mingyu Yang, Hongshan Guo, Lu Yang, Jun Wu, Rong Li, Ping Liu, Ying Lian, Xiaoying Zheng, Jie Yan, et al. Single-cell RNA-seq profiling of human preimplantation embryos and embryonic stem cells. *Nature Structural and Molecular Biology*, 20(9):1131, 2013.

[19] Luke Zappia, Belinda Phipson, and Alicia Oshlack. Splatter: simulation of single-cell RNA sequencing data. *Genome Biology*, 18(1):174, 2017.
